# rapmad: Robust analysis of peptide microarray data

**DOI:** 10.1186/1471-2105-12-324

**Published:** 2011-08-04

**Authors:** Bernhard Y Renard, Martin Löwer, Yvonne Kühne, Ulf Reimer, Andrée Rothermel, Özlem Türeci, John C Castle, Ugur Sahin

**Affiliations:** 1 The Institute for Translational Oncology and Immunology (TrOn), 55131 Mainz, Germany; 2 Research Group Bioinformatics (NG 4), Robert Koch-Institute, 13353 Berlin, Germany; 3 JPT Peptide Technologies GmbH, 12489 Berlin, Germany

## Abstract

**Background:**

Peptide microarrays offer an enormous potential as a screening tool for peptidomics experiments and have recently seen an increased field of application ranging from immunological studies to systems biology. By allowing the parallel analysis of thousands of peptides in a single run they are suitable for high-throughput settings. Since data characteristics of peptide microarrays differ from DNA oligonucleotide microarrays, computational methods need to be tailored to these specifications to allow a robust and automated data analysis. While follow-up experiments can ensure the specificity of results, sensitivity cannot be recovered in later steps. Providing sensitivity is thus a primary goal of data analysis procedures. To this end we created rapmad (Robust Alignment of Peptide MicroArray Data), a novel computational tool implemented in R.

**Results:**

We evaluated rapmad in antibody reactivity experiments for several thousand peptide spots and compared it to two existing algorithms for the analysis of peptide microarrays. rapmad displays competitive and superior behavior to existing software solutions. Particularly, it shows substantially improved sensitivity for low intensity settings without sacrificing specificity. It thereby contributes to increasing the effectiveness of high throughput screening experiments.

**Conclusions:**

rapmad allows the robust and sensitive, automated analysis of high-throughput peptide array data. The rapmad R-package as well as the data sets are available from http://www.tron-mz.de/compmed.

## Background

Peptide microarrays have emerged as a promising technique for the simultaneous high throughput analysis of peptide characteristics. Synthesized peptides are spotted in a grid-layout on glass slides which allow the screening of thousands of peptides within a single experiment with requiring only a small quantity of sample. Applications range from studying the humoral response to HIV [1] or food allergens [2] to the detection of cancer biomarkers [3] and antibody signatures [4] to the characterization of protein-protein interactions [5] and of kinase substrates [6,7].

While peptide microarrays offer enormous potential for a wide range of applications and while significant improvements have been made with regard to the reliable spotting of small amounts of peptides at closely neighboring, well-defined spatial positions [8,9], a major bottle neck remains in the automated analysis of the acquired data.

Numerous tools have been developed for the analysis of DNA microarray data, see e.g. [10,11] for reviews, but these cannot easily be transferred and require major adjustments. For instance, rather than quantifying the impact of differential expression, peptide microarrays experiments commonly only result in a single wavelength measurement per peptide. Usually, only a small proportion of the spotted peptides is expected to show a signal and requires reliable identification. Further specific challenges of the analysis of peptide microarray data include the diverse sources of noise, ranging from peptide synthesis artifacts to unspecific binding effects to peptides.

Existing tools specifically developed for the analysis of peptide microarray experiments can be categorized into three groups, graphical analysis tools, differential analysis tools, and general analysis tools.

As graphical tools, a combined approach of clustering and principal component analysis to visualize similarly behaving groups of peptides has been introduced [12] as well as an integrated webserver for the storage of peptide microarray experiment data with several graphical analysis steps [13].

For differential analysis, several approaches have adapted differential expression detection schemes to peptide microarrays, including a support vector machine driven webtool for distinguishing peptide binding intensities of two experimental groups [14] and adapted statistical tests for differentiating measured intensities for two populations [1,15].

The more general question of identifying signal carrying peptide spots and accounting for peptide microarray specific sources of noise has been addressed using a robust version of a z-score for the difference of the intensity of a specific peptide spot to empty spots to identify signal carrying peptide spots [2] and a linear model fit on all peptide spot measurements to account for several systematic effects [16]. This approach was extended by a signal calling step based on a t-test and a cutoff based removal of secondary binding spots [17].

Here, we introduce a novel method for the general analysis of peptide microarray data, that significantly extends the existing approaches. Using several classes of control peptides, we apply a linear model for normalization and the removal of systematic array effects. Further, we use a mixture-model to identify secondary antibody binding peptide spots and apply a probabilistic approach for signal calling which does not rely on arbitrary thresholds, but provides a slide specific estimate. Additionally, we provide a machine-learning driven quality control procedure to computationally exclude intensity measurements of low reliability.

After describing our methods in detail, we apply it to data from a cancer-biomarker detection study and demonstrate improvements relative to the existing general analysis tools, particularly with regards to sensitivity.

## Method

### Peptide Microarrays

**Figure 1:**
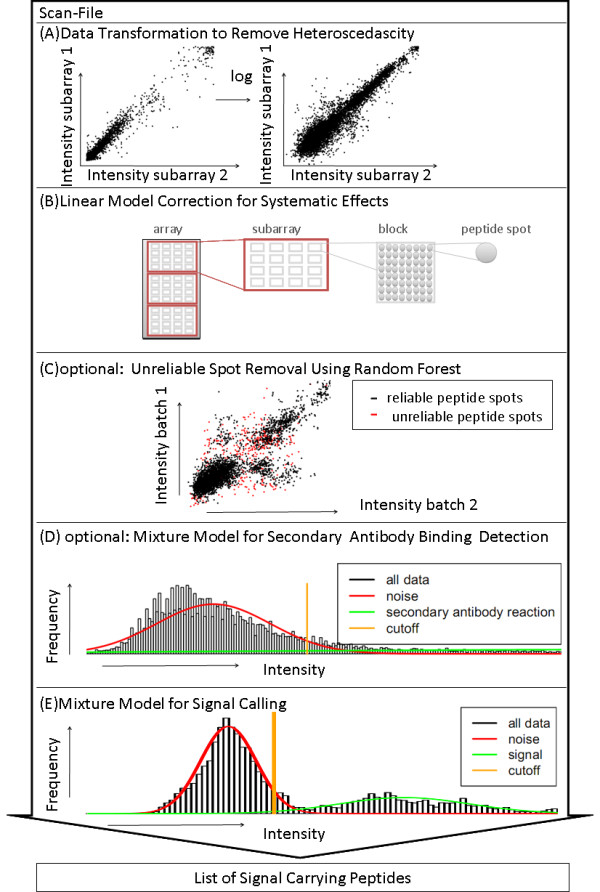
**Flowchart**. Flowchart of the data analysis pipeline for extracting a list of signal-carrying peptides from the measured intensities of a peptide microarray scan.

The layout of the microarray slides used in this study is based on a three level hierarchy (Figure 1B): Each array (i) consists of three subarrays (ii) which are identical in terms of individual peptide placement but are printed consecutively; thus, each individual peptide is spotted as a triplicate. Each subarray has 16 blocks (iii) which are arranged in a four by four pattern; each of these blocks is printed by a single print tip. Each block has 20 rows and 20 columns, resulting in 19200 peptide spots per array.

Printed slides are scanned at 532 nm wavelength for quality control, with peptide spots being visible due to scatterlight effects. In brief, the incubation experiment and image acquisition consist of the following steps: (i) blocking of the array surface to reduce unspecific binding, (ii) incubation of the slides with sample material, (iii) incubation with a fluorescently labeled secondary antibody and (iv) scanning at 635 nm wavelength and spot detection. We refer to a peptide as being reactive if we observe fluorescence of the labeled secondary antibody indicating that primary antibody of the sample bound to the peptide. For a detailed protocol of microarray incubation experiments including the motivation of acquisition conditions see [9].

It is noteworthy that our computational methods described in the further sections accept generic data as input and are not restricted to the array layout and procedures mentioned above.

### Algorithmic Overview

Figure 1 shows an overview of the individual steps of the workflow. Following the read out of the raw data of a scanned peptide microarray, we apply a transformation to reduce the heteroscedasticity (Figure 1A). Using a linear model fit on selected control peptides, we remove array effects (Figure 1B). In an optional subsequent step, we can exclude unreliable spots using a machine learning procedure (Figure 1C). Finally, we apply a mixture model to identify peptides binding to the secondary antibody used for coloring (Figure 1D), before providing a probabilistic signal call (Figure 1E).

The analysis pipeline is implemented in R [18] and available from http://www.tron-mz.de/compmed.

### Read Out and Transformation of Raw Data

To transform an image file from the scan of a peptide microarray to individual intensity values for each single peptide, we apply GenePix Pro, version 7 (Molecular Devices, Sunnyvale, CA, USA), using the mean foreground intensity (without background subtraction) from the GenePix Result (gpr) file as a measure for intensity.

It has been previously described [2,16] that peptide microarray measurements show strong heteroscedasticity with larger variances for high intensity measurements. By applying a binary logarithm transformation to each peptide spot mean intensity, we stabilize the variance allowing the application of a linear model procedure.

### Control Peptide Based Linear Model Fit

Peptide microarray intensity measurements can be perturbed by a variety of factors, some of which have been proposed in the model of [16]. These factors include array-to-array effect (*A_i_*) resulting from the printing process or storage conditions. Further, depending on the spatial position of a peptide spot, it might be incubated with a different amount of sample. This relates to the overall row (*R_j_*) and column (*C_k_*) position of a spot as well as to the subarray (*S_l_*) to which a spot belongs. Further, variation might be introduced by the printing needle (*N_m_*) used. The effect of a peptide itself is then denoted by *P_n_*. Thus, an intensity measurement *Y_ijklmn _*can be modeled as(1)

With the variance stabilizing logarithm transformation applied in the previous step, the residuals (*ε_ijklmn_*) can be assumed to follow a normal distribution with constant variance. In most applications, the number of repeats per peptides are very limited and the placement of peptides is not in random order, but rather defined by practical considerations of the printing process. Thus, confounding of effects can arise, for instance the placement of predominantly strongly reacting peptides at positions spotted with one needle might result in the wrongful attribution of a strongly positive needle effect. To avoid these confounding effects, we limit the fitting of the linear model of equation 1 to a set of control peptides, which are printed with high number of repeats for each needle and subarray to allow reliable estimation. While this limitation renders the estimation of a peptide effect *P_n _*infeasible, all remaining coefficients can be reliably estimated and can be used to correct all peptide measurements. This allows us to estimate the parameters *A_i_, R_j_, C_k_, S_l_*, and *N_m _*for all values of *i*, *j*, *k*, *l*, and *m*. We then correct all measurements, for which we could not estimate the peptide specific effect *P_n_*, by computing .

### Unreliable Spot Removal

When historic data is available for peptide arrays with identical slide layout, this data can be used to remove unreliable spots. Since this is not possible in all setups, we regard this as an optional step for improving and robustifying peptide microarray data analysis.

We apply a random forest procedure [19] to identify potentially unreliable peptide spots [20]. Unreliable spots may arise from a variety of not explicitly modeled experimental effects, e.g. the different decay of spotted peptides, dust effecting peptide spots or errors in the alignment of spots by software such as GenePix. These variation can drastically reduce the reproducibility of peptide array experiments and may result in misleading conclusions.

Using historic data from previous experiments with slides of an identical layout, we train a random forest classifier to detect spots which are not reproducible between subarrays. A random forest classifier is a machine learning procedure, which learns label categories from existing data and then applies them to new, previously unclassified data sets. It shows good predictive power while being robust to parameter setting [21] and favorable theoretical properties (e.g. with regard to overfitting [19]).

For the training of the classifier, we use the fact that each peptide is repeated on various subarrays (in our case three). We label all subarray spots which are not within a 95% linear regression confidence band for a single linear regression on either of the remaining two subarray repeats as unreliable and all other spots as reliable. We use all columns of the result file besides the sequence as well as all columns of the quality control result file of a corresponding scatterlight scan for each spot as explanatory variable (with a column representing a property of a peptide spot, i.e. pixel intensity variation or spot size; see additional file 1 for a complete listing). We use random sampling to balance the set of unreliable and reliable spots in their size and then use the prediction of the random forest to label all spots of a new data set. For each peptide, we directly discard all those spots indicated as unreliable and only average over the remaining spots, weighted by their probability of being reliable. If all spots of one peptide are marked as unreliable, we exclude this peptide from any further analysis.

### Mixture Model for Secondary Antibody Binding Detection

In peptide microarray experiments, secondary antibody binding peptides pose a major complication. When peptides show a direct reaction with the dye-coupled secondary antibody, two effects are confounded. It cannot be distinguished to which extent a measured intensity is explained by this undesired and unspecific reaction rather than the specific reaction of interest. Thus, to avoid ambiguous results which hinder interpretation and may result in misleading conclusions, peptides showing significant unspecific reaction with the secondary antibody need to be identified and excluded from further analysis. Reactivities on empty slides without any sample have been identified as important indicators [17]. In experimental settings not allowing empty slides, this step can be excluded from the overall data analysis pipeline with an increased risk of false positives. However, this approach is tailored to identifying the unspecific binding of the secondary antibody to the peptide, not to the slide itself, which should affect all spots in a similar mode and thereby increase the noise level. This effect is addressed by the subsequent signal calling step. Rather than relying on a fixed threshold, we propose a mixture model strategy to derive a probabilistic criterion for the identification of secondary antibody binding peptides from empty slides. For this purpose, we assume that the normalized intensities of all peptides on the empty slides stem from two populations: one population for peptides, which do not show any reaction to the secondary antibody besides noise and a second population for peptides which show a reaction to the secondary antibody. We model each population by a normal distribution and let the mean and variance of the first population be denoted by *μ*_1 _and  and for the second population by *μ*_2 _and . A schematic display of a histogram of the measured intensities and the two underlying distributions in red (noise) and green (reaction to secondary antibody) is given in Figure 1D. The orange line indicates the resulting cutoff.

We apply an expectation maximization algorithm [22,23] to iteratively adapt the estimates for *μ*_1_, *μ*_2_, , and , and for each measured normalized intensity, we update the likelihood that it belongs to either one of the two populations [24,25]. For the initialization, we rely on the empirical mean and variance estimate for two groups of control peptides. One group for which we do not expect any reaction with the secondary antibody and a second group of peptides which are known to show various degrees of reactivity.

We then use the resulting distribution of the peptides which do not show any reaction to the secondary antibody, to derive a criterion for secondary antibody binding. Since we expect most peptides not to show any reaction, we expect this distribution to be more reliably estimated and mostly driven by random variations. We regard the upper 5% quantile  of this distribution to be the cutoff. Thus, for any measured normalized intensity on the empty slides , we compute(2)

Any resulting value of  below 0.05 indicates only low likelihood for this intensity being this large by chance [24] and thus the corresponding peptide is disregarded for the further analysis. With this cutoff generation we rely on the assumption that at least the population of peptides not showing a secondary antibody binding follow a normal distribution. However our historic slides at least approximately support this assumption (see additional file 1).

Instead of this cutoff generation, it is also possible to directly compute a false discovery rate from the likelihood of the mixture model components (and our code allows this option), however this relies more heavily on the correctness of the normality assumption for the group of the reactive peptide than the proposed approach, which depends more on the not reactive peptides, in which we are more confident.

### Mixture Model for Signal Call

To distinguish those peptide spots which carry a signal from those spots which only display noise, we again apply a mixture model. We assume that both, the signal-carrying peptide spots as well as the noise spots can be modeled by normal distributions with mean *μ*_signal _respectively *μ*_noise _and standard deviations  respectively . A schematic display of a histogram of the measured intensities and the two underlying distributions in red (noise) and green (signal carrying peptides) is given in Figure 1E; the orange line indicates the resulting cutoff. We initialize these parameters again based on suitable control classes. For one class of peptides expected to show a reaction, and for a second class of peptides not expected to show a reaction, we separately compute the empirical mean and variance and use these as starting values. We then update these values as well as the likelihood for each data point using the expectation maximization algorithm as described for the secondary antibody binding detection.

We then use the distribution of the noise spots to identify the signal carrying spots which depart significantly from the noise distribution by identifying the upper 5% quantile  of the noise distribution as a cutoff. Thus, for any measured normalized intensity , we compute  analog to equation 2. Any resulting value of  below 0.05 indicates only low likelihood for this intensity being this large by chance.

### Experiments

Experiments are conducted on a total of 115,200 peptide spots which were printed on six PepStar peptide microarrays (JPT, Berlin, Germany) with an identical design and printed in two batches (print batch 1 and print batch 2). Two separate batches were chosen to show the maximum possible inter-experiment variation. On each slide 10% of the total 19,200 spots are used as dedicated control spots. 5% of all peptides are meant as positive controls, expected to show strong specific reactions, 2.5% are secondary antibody controls, which are expected to show strong reactions with the secondary antibody. 1% of all peptides are negative controls not expected to show any reaction and the remaining 1.5% are process controls. Among the remaining 17,280 peptide spots per array, 666 spots contain peptides of human PLAC-1 (UniProt accession Q9HBJ0) and 513 spots contain peptides of NYESO, VEGFA, and CD20 (UniProt accession P78358, P15692 and P11836, respectively; all of human origin). All proteins are represented by peptides with a length of 15 amino acids and an overlap of 11 amino acids. The slides were incubated in a HS 4800 Pro hybridization station (Tecan, Maennedorf, Switzerland). One slide per print batch was incubated with diluent only (empty slide), one slide per batch with a pooled plasma of six patients without indication of cancer and low concentration (1 ng/ml) of a PLAC1-specific spike-in antibody (Michael Koslowski, personal communication) and the remaining slide per batch with the same sample material, but high concentration (3 ng/ml) of the PLAC1-specific spike-in antibody. The experimental setup is summarized in table 1. A Cy5-conjugated AffiniPure Mouse Anti-Human IgG (H+L) (Jackson Immuno Research Laberatories, Avondale, PA, USA) was used as a secondary antibody. All slides were scanned with a GenePix 4300 microarray scanner (Molecular Devices, Sunnyvale, CA, USA) and converted to a result file using GenePix Pro, version 7 (Molecular Devices, Sunnyvale, CA, USA). All result files are available from the authors' website.

**Table 1:** Experimental Setup

Array number	Print batch	Spike-in antibody concentration [ng/ml]	Plasma
1	1	-	-
2	1	1	+
3	1	3	+
4	2	-	-
5	2	1	+
6	2	3	+

Summary of the experimental setup for the six microarrays used in this study.

The analysis of results is focused on five aspects: We evaluate (i) the efficiency of the linear model in reducing the unexplained variance, (ii) the unreliable spot finding procedure by comparing the reproducibility of the two print batches before and after quality control, (iii) the removal of secondary antibody binding peptides by focusing on the NYESO, VEGFA, and CD20 peptides, which are not expected to show any reaction with the given normal sera, with the exception of secondary binding effects, and (iv) the efficiency of the signal call by analyzing the specificity and sensitivity of our approach based on the NYESO, VEGFA, and CD20 peptides which are expected to be non-reactive and the PLAC1-peptides, which we expect to show a reaction. We regard NYESO, VEGFA, and CD20 peptides showing a reaction as false positives (*fp*) and those not showing a reaction as true negatives (*tn*).

Similarly, we regard PLAC-1 peptides not showing a reaction as false negatives (*fn*) and those showing a reaction as true positives (*tp*). Sensitivity, specificity, and accuracy are then given by(3)

In a final step (v), we compare our approach to the approaches introduced by [2] and [17] based on the accuracy.

To judge the significance of the results of experiments (iii)-(v), we computed 95% bootstrapped confidence intervals for all sensitivity, specificity and accuracy values based on 1000 times resampling from the peptide intensities of each class. In order not to confound the results of the unreliable spot finding step (ii) with the subsequent steps and to allow the fair comparison with the existing methods which do not identify unreliable spots, experiments (iii)-(v) were run on all peptide spots, even though some were marked as unreliable.

## Results

We analyze the effect of the major steps of our approach including the linear model, the quality control and the secondary antibody binding detection and signal call as well as comparing the overall procedure on the antibody reactivity data.

With regard to the linear model fit, a summary of results for print batch 1 and the high spike-in antibody concentration is given in table 2. It is evident that the peptide effect itself is the major influence of the measured intensity. Still, all remaining explanatory variables, which model systematic effects, are highly significant as shown by the p-values for the corresponding F-Test. Row and column effects, so the spatial position of a peptide, show the overall strongest influence of the systematic effects. The needle and subarray effect are smaller, but still highly significant. However, it should be noted that a confounding of effects cannot be excluded, e.g. when considering a single needle only a subset of row and column positions are addressable on a subarray. Including all interaction effects requires an unfeasibly high number of degrees of freedom, while choosing subsets does not significantly improve results (data not shown). Overall, the systematic effects contribute to reducing the unexplained variation in the measured peptide intensity data by 65%.

**Table 2:** Linear model fit summary

	Df	Sum Sq	Mean Sq	F value	Pr(>F)
Peptide	12	170916	14243.0	57461.65	< 2.2e-16
Subarray	2	24	12.2	49.18	< 2.2e-16
Needle	15	45	3.0	12.08	< 2.2e-16
Row	234	458	2.0	7.89	< 2.2e-16
Column	75	409	5.5	22.03	< 2.2e-16
Residuals	2218	550	0.2		

Linear model fit summary. The F-values and the corresponding probabilities (Pr(>F)) clearly indicate that all explanatory variables used are highly significant and that each contributes to reducing the variance present in the arrays. The peptide sequence itself shows by far the strongest effect while requiring only twelve degrees of freedom (Df), it shows a sum of squares for its effect (Sum Sq) of 170,916, much stronger than the residuals sum of squares of 550. While not as strong as the peptide effect, the remaining explanatory effects reduce the residuals by 65%. Column and row effects are strongest, but subarray and needle effects require fewer degrees of freedom, resulting in strong mean sum of squares (Mean Sq) values.

**Figure 2:**
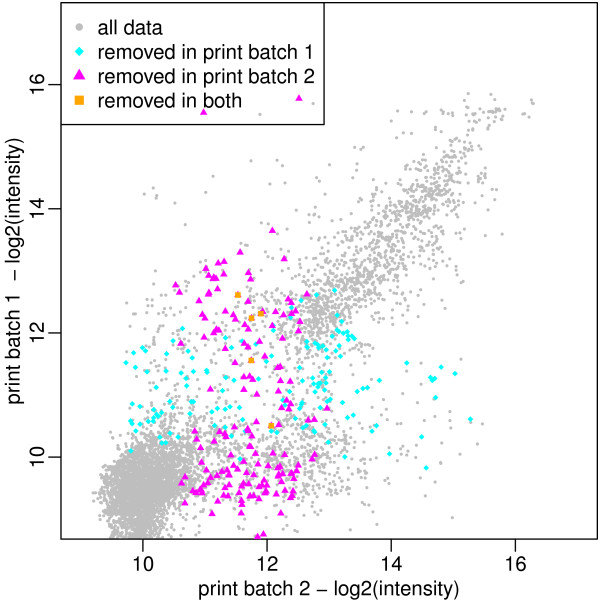
**Unreliable Spot Finding**. Scatter plot of intensities from slides of two print batches with the same high antibody concentration on a binary logarithm scale. Due to experimental noise, we see departures from the diagonal line on which we would expect all data points. The quality control algorithm identifies approximately 2.5% of all data points in each print batch as unreliable across all subarrays; these peptides are removed accordingly (colored in magenta, cyan and orange), resulting in an increase of the coefficient of variation of approximately 3%. While not identifying all outlying observation, the removed spots primarly affect peptide spots which show large variation between the print batches.

After training on a total of 8 historic slides with identical layout, the unreliable spot finding procedure marks 8.3% of all spots in print batch 1 and 11.8% of all spots in print batch 2 as unreliable for the high concentration experiment. Since peptides are excluded only in cases when all three replicates for one peptide are marked as unreliable, this results in the removal of 2.4% of all peptides in print batch 1 and 2.6% of all peptides in print batch 2. Visual inspection (cf. Figure 2) shows that these peptides primarily show large variation between the two data sets. By removing these peptide spots, we see an improvement of 3% in the coefficient of variation from *R*^2 ^= 0.72 to *R*^2 ^= 0.75. Still, not all outliers are detected by the approach. Analysis of the missed outliers however indicates that these undetected outliers consistently affect replicates of only nine different peptide sequences, in contrast to the 129 different peptide sequences marked as unreliable by the unreliable spot finding procedure.

We evaluated our secondary antibody binding removal approach by computing the number of peptides which were excluded from the further analysis because of secondary antibody removal. Out of 5760 non control peptides 354/501 (print batch 1 and print batch 2, respectively) were identified with reaction on the empty slides and excluded from the further analysis. This affected none of the PLAC-1 peptides which were expected to show a specific reaction and 0/2 (print batch 1 and print batch 2, respectively) of the NYESO, VEGFA and CD20 peptides not expected to show a reaction. Consequently, when regarding the sensitivity and specificity which were computed based on these peptides, we observe that with the secondary antibody binding removal the specificity is slightly increased while the sensitivity remains unchanged when compared to running our approach without this step.

**Figure 3:**
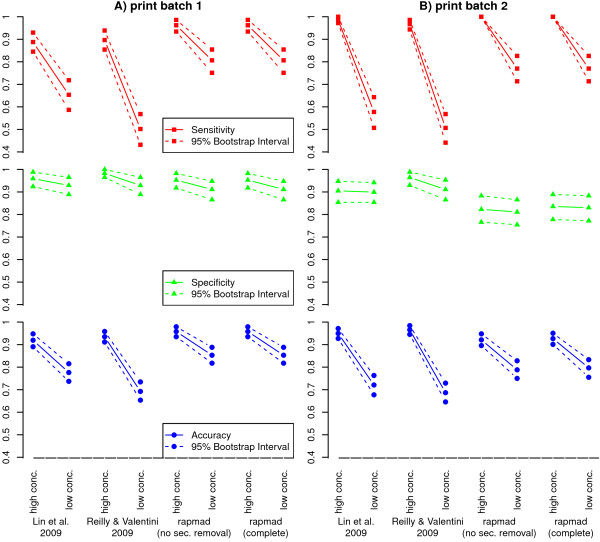
**Sensitivity, Specificity and Accuracy in Comparison**. Sensitivity, specificity and accuracy for our approach without and with secondary antibody binding removal in comparison to the approaches of [2] and [17]. Both, the high (3 ng/ml) and the low (1 ng/ml) spike-in antibody concentration slides were evaluated for both print batches (left and right). 95% bootstrap confidence intervals were computed based on 1000 times resampled peptide intensities and are shown by dashed lines. For all approaches, the specificity remains rather constant when reducing the spike-in antibody concentration, while we see a general decline in sensitivity and accuracy. For the approaches of [2] and [17] the decline in accuracy is rather steep, our approach shows still good accuracy above 0.8 for the low antibody concentration.

The sensitivity and specificity for our approach are shown in Figure 3 in comparison to the approaches of [2] and [17]. For two print batches, two slides were incubated with a low and a high concentration of spike-in antibody, respectively. Overall, we observe for all approaches that sensitivity is more affected by the reduction of the antibody concentration than specificity, which is rather stable. In comparison, our approach shows best sensitivity across all settings. It shows a sensitivity of 0.96 to 1.00 compared to 0.89 to 0.98 for the other two approaches for the high antibody concentration data. This difference is more expressed for the low antibody concentration data with a sensitivity of 0.77 to 0.81 for our approach compared to 0.50 to 0.65 for the previous two approaches. Specificity is slightly worse for our approaches for print batch 1 with values of 0.91 to 0.95 for our approach and 0.92 to 0.98 for the existing approaches. Similarly for print batch 2, our approach features specificity of 0.83 to 0.84 compared to 0.89 to 0.96. As a consequence of the substantially improved sensitivity, our approach also displays best accuracy. While this difference is small for the high antibody concentration with accuracy values of 0.93 to 0.96 for our approach compared to 0.92 to 0.96 for the previous approaches, it is much more substantial for low antibody concentration. Here, our approach shows an accuracy ranging from 0.80 to 0.85, compared to 0.68 to 0.78 for the two other approaches.

rapmad is based on the assumption of (approximate) normality, especially for the linear model fit and the mixture model. For all arrays incubated with plasma, we analyzed these assumptions and find support for approximate normality (see additional file 1).

## Discussion

### Removal of systematic bias

By applying a linear model which explains 65% of the noise variation in the data, rapmad significantly improves the reliability of peptide microarray experiments. In addition to previous findings [16], we establish that also the spatial position on the peptide microarray is of high impact for removing systematic effects. This is important for distinguishing low intensity signals from noise. Since the effects are of a systematic nature (e.g. a decline in signal intensity associated with spatial position), it also reduces the risk that systematic effects are mistakenly identified as peptide signal effects. Despite the strong reduction in unexplained variation, not all occurring systematic effects are necessarily included in the model. For instance, we decided against including all interaction effects. While it can be of high interest to model the interaction of a specific peptide with a specific needle, there is usually not a sufficient number of replicates to allow a reliable estimation in a standard experiment. However, for large-scale experiments, it could be beneficial to design specific slides with sufficient replicates to quantify these effects. The effects of the secondary antibody are accounted for within subsequent analysis steps and are thus not modeled explicitly.

### Improved data quality after removal of unreliable measurements and secondary binding peptides

The production of peptide arrays as well as the binding of the secondary antibody involve sophisticated chemical processes. In spite of numerous experimental improvements [8,9] and constant monitoring, artifacts resulting from errors in the spotting of peptides or the unspecific binding of the secondary antibody can strongly influence measurements and result in misleading interpretations. We have proposed two computational statistics driven approaches to identify and exclude these artifacts and show that they can be successfully applied.

By marking approximately 2.5% of the peptide spots as unreliable, we can improve the reproducibility and thus the data quality. It should be noted however that still not all unreliable and irreproducible peptides are detected. The small number of peptide sequences being connected to the undetected outlying observations show that this entails a sequence-specific phenomenon rather than a print process artefact which would affect all sequences more evenly. While characteristics of the print process are captured within the available learning data, for instance in the scatterlight scans, sequence-specific effects are difficult to detect. In addition, the unreliable peptide spot finding procedure is limited by the available training data. We rely on labels based on the assumption that a specific replicate which departs from the remaining two replicates should be labeled as unreliable. We are aware that this definition is not necessarily correct since there are cases when the two remaining replicates are unreliable, but the specific replicate itself is correct. This can result in incorrect labels, which strongly reduce the quality of the random forest prediction. While manually validation could improve the label-quality, this reduces the applicability of the procedure to other data than our own. Thus, we accept a possibly smaller impact of the quality control procedure to allow the fully automated adaption to other datasets. Historic data for training is not always available and thus, this is an optional step for our pipeline.

By identifying 1.2% of the spots as secondary antibody binding, we can improve the specificity of the procedure without sacrifying its sensitivity. While the impact on the data shown here is comparatively small, this step can be of key importance to avoid wrongful interpretation.

When dealing with peptides prone to printing or synthesis problems or the binding of secondary antibody to peptides, any data analysis tool is challenged by the fact that the relevant information was lost before data acquisition, rendering the complete recovery of the information impossible. While not recovering the correct information, the magnitude of excluding wrongful results is significant. If wrongful results were not excluded, research could result in wrongful conclusions. With the removal of wrongful results no conclusions are possible for these doubtful peptide spots. Since in most cases, only subsets of peptide measurements are affected and designs usually contain several replicates, repeats of experiments are often not required to come to reliable conclusions.

### Strong sensitivity for low intensities

A particular strength of rapmad is the fact that it is especially suitable for low intensity measurements such as those occurring for low antibody concentrations. While the problem of separating signal from noise is substantially more difficult when the signal intensity is reduced, we only see a comparatively minor reduction in accuracy when reducing spike-in antibody concentration. Sensitivity is affected more significantly than specificity, but still at or above 0.8.

This robustness of our approach can be explained by the adaptivity of the signal calling procedure, which is based on correctly estimating the noise distribution and generating a noise-threshold based on this distribution. Thereby, it is less affected by changes in the signal intensity. As a consequence, the noise-threshold is adaptive and even low intensity signals can be detected to be above the threshold, resulting in a strong sensitivity.

### Favorable comparison to existing approaches

The strength of rapmad to maintain a high sensitivity even for low intensity settings is also what sets it apart from existing algorithms. Thus, the sensitivity and accuracy of our approach in low antibody concentration experiment is competitive to the ones of the two other approaches for high antibody concentrations. This is a result of the adaptive procedures which are better suited to distinguish signal from noise in low intensity situation than preset thresholds and demonstrates that our computational approach is capable of compensating for experimental limitations. For high intensities as seen with high antibody concentration, it is generally easier to achieve high sensitivity due to a better separation of signal and noise in the raw data. That is also why we see less of a benefit of rapmad in comparison to existing algorithms.

### Increasing the effectiveness of screening procedures

Peptide microarrays have been primarily used within screening settings with follow-up experiments focusing on a small number of promising candidates to ensure specificity [4]. Unidentified reactive peptides from a peptide microarray experiment cannot be distinguished from the large number of rightfully excluded unreactive peptides and are thus not further regarded. By substantially increasing the sensitivity for low intensity reactions, our approach avoids the wrongful exclusion of peptides and thereby increases the effectiveness of screening procedures. While standard settings were used in all experiments, rapmad additionally allows the user to adapt the quantiles used in the mixture models to trade additional sensitivity for specificity.

## Conclusion

Within this contribution, we introduce rapmad, a tool for the robust and rapid analysis of peptide microarray data. rapmad is an automated, multi-step approach that combines several computational statistics procedures to augment the data quality of peptide microarray and to allow a reliable analysis. Its steps include the preprocessing of the data by removing systematic effects, the exclusion of unreliable measurements and secondary antibody binding peptides, and a probabilistic signal call for reactive peptides.

In comparison with existing algorithms, it shows competitive performance for high antibody concentration. For low antibody concentration, it shows a significant increase in accuracy over existing approaches. This is mainly due to its substantially improved sensitivity at competitive specificity.

With its increased sensitivity and its automated data analysis, rapmad can thereby contribute to establish and broaden the usage of peptide microarrays as a standard tool for a wide-range of peptidomics applications.

## Authors' contributions

BYR, ML, UR, JCC, and US designed the methods, BYR and ML wrote code and analyzed data. All authors contributed to the design of the experiments. YK and AR set up and ran the experiments. BYR wrote the manuscript with contributions from ML and JCC. All authors read and approved the manuscript.

## Supplementary Material

Additional file 1 **Supplementary Figures**. PDF document containing supplementary figures. Click here for file
