# Supplementary material for: rapmad: Robust analysis of peptide microarray data
Source: BMC Bioinformatics. 2011 Aug 4;12:324. doi: 10.1186/1471-2105-12-324 (PMC3174949; doi:10.1186/1471-2105-12-324)
Supplement: Additional file 1 — Supplementary Figures. PDF document containing supplementary figures. [file 1471-2105-12-324-S1.PDF]

# Supplementary Material: rapmad: Robust Analysis of Peptide Microarray Data

Bernhard Y Renard[1,2,‡], Martin Löwer[1,‡], Yvonne Kühne[1], Ulf Reimer[3], Andrée Rothermel[1], Özlem Türeci[1], John C Castle[*,1], Ugur Sahin[1]

[1]The Institute for Translational Oncology and Immunology (TrOn), 55131 Mainz, Germany
[2]current address: Research Group Bioinformatics (NG 4), Robert Koch-Institute, 13353 Berlin, Germany
[3]JPT Peptide Technologies GmbH, 12489 Berlin, Germany
[‡]authors contributed equally

Email: Bernhard Y Renard - renardB@rki.de; Martin Löwer - martin.loewer@tron-mainz.de; Yvonne Kühne - yvonne.kuehne@tron-mainz.de; Ulf Reimer - reimer@jpt.com; Andrée Rothermel - andree.rothermel@tron-mainz.de; Özlem Türeci - oezlem.tuereci@tron-mainz.de; John C Castle*- john.castle@tron-mainz.de; Ugur Sahin - ugur.sahin@tron-mainz.de;

[*]Corresponding author

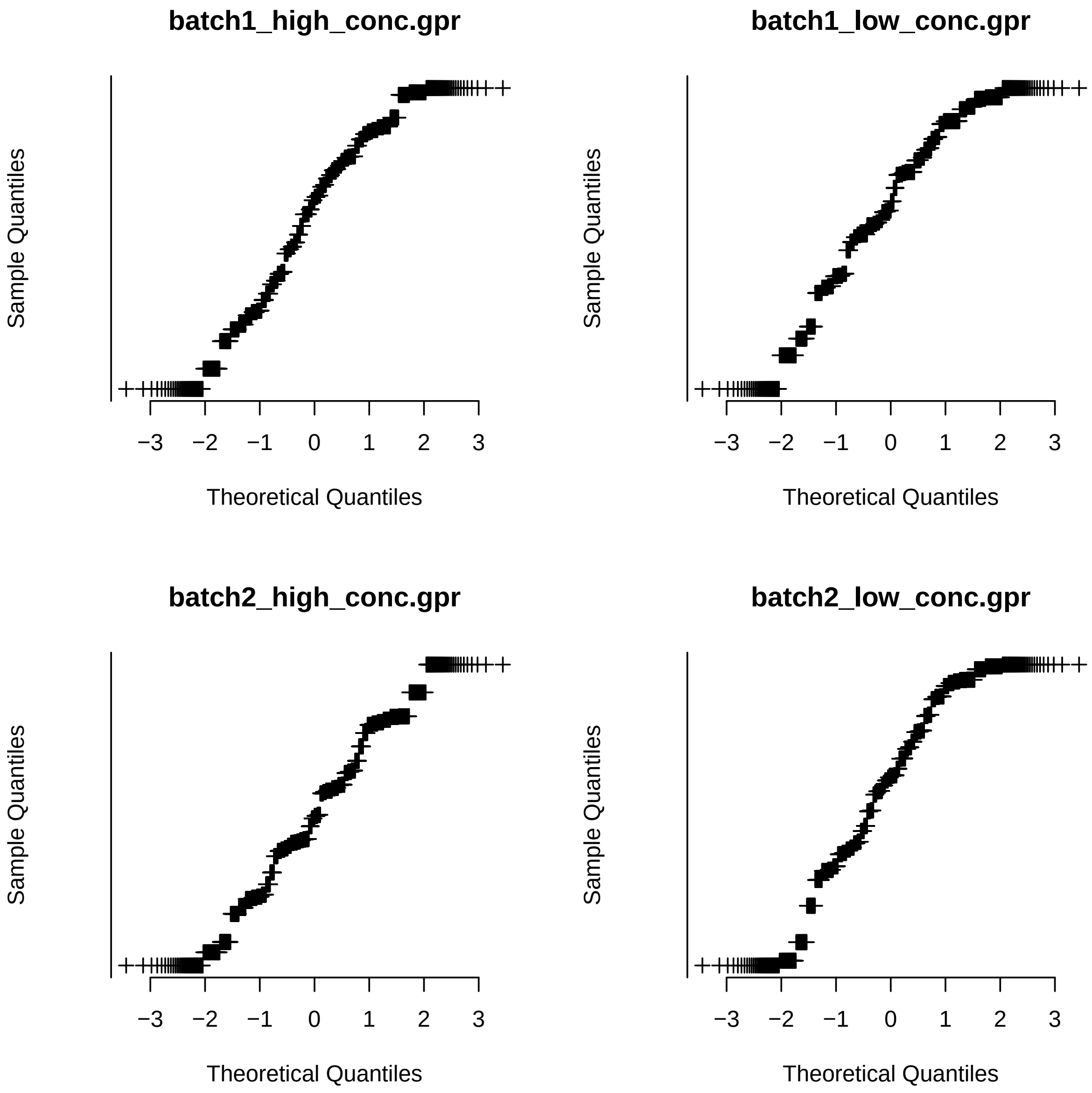


Figure 1: Quantile-Quantile-Plots for assumption checking. For all datasets incubated with plasma (cf table 1), a quantile-quantile plot of the residuals is shown. While we see departures from a straight line (and normality) towards the tails of the distribution, we observe an acceptable fit within the center and no clear evidence against the normality assumption.

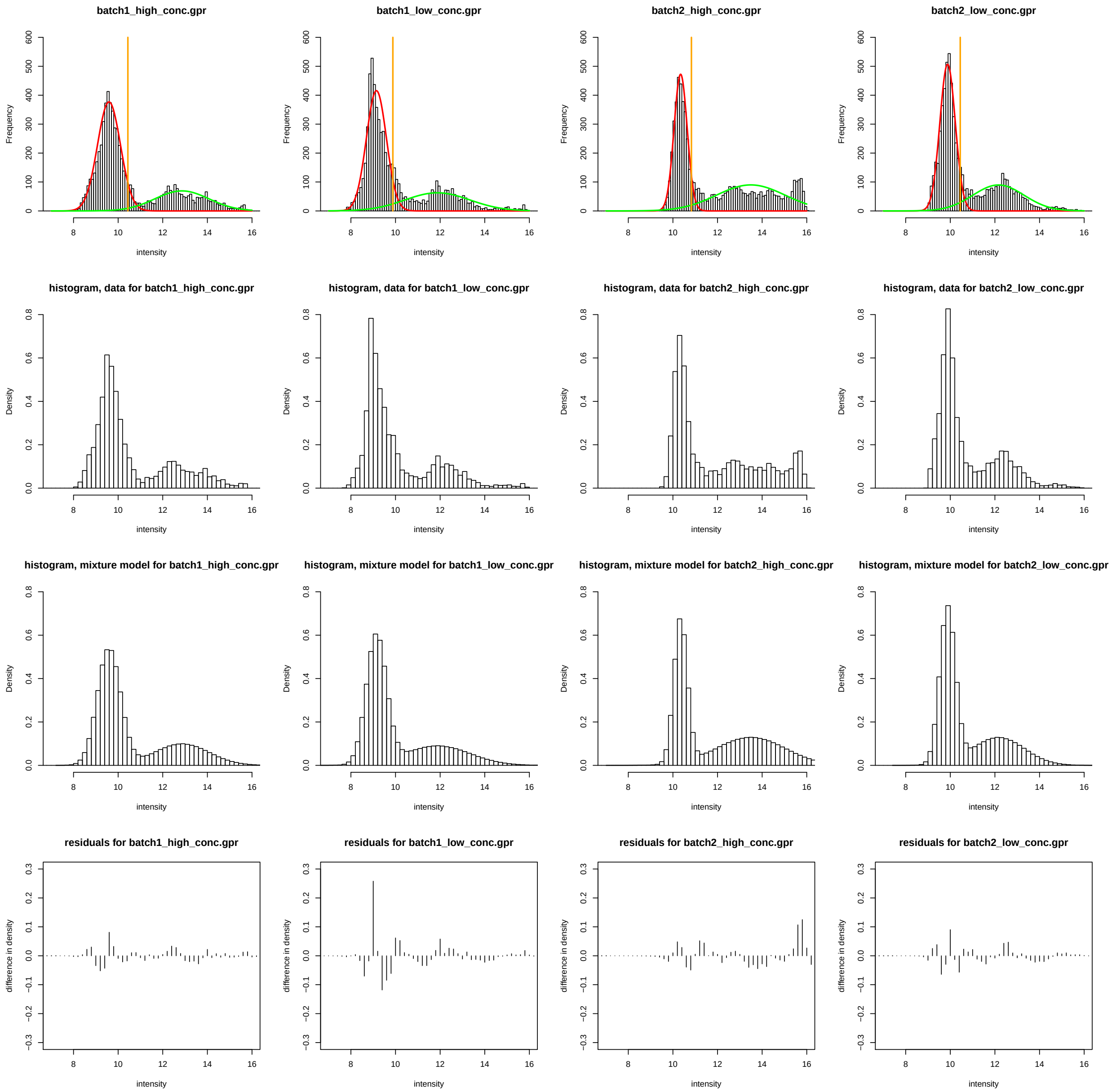


Figure 2: Mixture Model Evaluation Plots. Each column shows the results for one of the data sets incubated with plasma (cf table 1). The first first column shows the histogram of the intensity data and in red the normal distribution for the 'noise' distribution and in green the model fit for the 'signal' distribution. The vertical orange line shows the chosen cutoff. The second row shows only the histrogram for the raw data whereas the third row shows the histogram for the mixture model (so the mixture of normal distributions we assume). Finally, the fourth line shows the residuals between the raw data and the assumed mixture model. Overall, we see an acceptable model fit, especially for the 'noise' distribution. However, we see more limited support for the 'signal' distribution. This can impact the reliability of a false discovery generation computation, but our cutoff generation is primarily based on the 'noise' distribution and should thus be more robust.
